# Population‐specific responses of an insect herbivore to variation in host‐plant quality

**DOI:** 10.1002/ece3.8392

**Published:** 2021-11-24

**Authors:** Josephine Kuczyk, Ange Raharivololoniaina, Klaus Fischer

**Affiliations:** ^1^ Zoological Institute and Museum University of Greifswald Greifswald Germany; ^2^ Department of Biology Institute of Integrated Natural Sciences University of Koblenz‐Landau Koblenz Germany

**Keywords:** anthropogenic climate change, genotype by environment interaction (G × E), indirect effects, pace of life, phenotypic plasticity, *Pieris napi*

## Abstract

Anthropogenic climate change poses a substantial challenge to many organisms, to which they need to respond to avoid fitness reductions. Investigating responses to environmental change is particularly interesting in herbivores, as they are potentially affected by indirect effects mediated via variation in host‐plant quality. We here use the herbivorous insect *Pieris napi* to investigate geographic variation in the response to variation in food quality. We performed a common garden experiment using replicated populations from Germany and Italy, and manipulated host quality by growing host plants at different temperature and water regimes. We found that feeding on plants grown at a higher temperature generally diminished the performance of *P*. *napi*, evidenced by a prolonged development time and reduced larval growth rate, body mass, fat content, and phenoloxidase activity. Genotype by environment interactions (G × E) were present in several performance traits, indicating that Italian populations (1) respond more strongly to variation in host‐plant quality and (2) are more sensitive to poor food quality than German ones. This may reflect a cost of the rapid lifestyle found in Italian populations. Consequently, German populations may be more resilient against environmental perturbations and may perhaps even benefit from warmer temperatures, while Italian populations will likely suffer from the concomitantly reduced host‐plant quality. Our study thus exemplifies how investigating G × E may help to better understand the vulnerability of populations to climate change.

## Graphical abstract

Warm‐adapted populations of the butterfly *Pieris napi* responded more strongly to variation in host‐plant quality and were more sensitive to poor food quality than cold‐adapted ones. Consequently, cold‐adapted populations may be more resilient against environmental perturbations. Our study exemplifies how investigating G × E may help to better understand the vulnerability of populations to climate change.

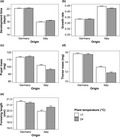

## 1 INTRODUCTION

Climate change causes rising mean temperatures and increasing frequencies of extreme weather events such as heat waves, severe drought, and heavy rain (Hansen et al., 2012; Rahmstorf & Coumou, 2011). Organisms are forced to respond to these changes in order to survive, as climate change may otherwise push them beyond their critical limits (Harvey et al., 2020; Soroye et al., 2020). Concomitant responses to novel environmental conditions may include phenotypic plasticity and genetic adaptation (Rodrigues & Beldade, 2020; Sgrò et al., 2016). Regarding the latter, populations often exhibit traits that provide an advantage under the specific environmental conditions encountered, termed local adaptation (Kawecki & Ebert, 2004). Consequently, populations often differ in fitness‐related traits across their geographical range, which is thought to reflect spatial variation in environmental conditions and corresponding selective pressures (Chown & Gaston, 2010; Posledovich et al., 2014; Sambucetti et al., 2006). A prominent example comprises variation in body size in insects. This may result from Bergmann size clines (larger body size in cooler environments), counter‐gradient variation (smaller body size in cooler environments), or differences in season length (with shorter growing seasons resulting in smaller body size; Blanckenhorn & Demont, 2004; Chown & Gaston, 2010; Nygren et al., 2008). Also, plastic responses to environmental variation are common. For instance, heat tolerance is well known to be environment dependent, increasing under warmer conditions and vice versa (Fischer & Karl, 2010; Franke et al., 2019; Günter, Beaulieu, Freiberg, et al., 2020). Finally, such plastic responses may differ among species and populations, reflecting genotype by environment interactions (G × E) (Günter, Beaulieu, Freiberg, et al., 2020; Saltz et al., 2018).

The existence of G × E is a fundamental concept in evolutionary biology, indicating genetic variation in trait plasticity (Oomen & Hutchings, 2015; Saltz et al., 2018). As a consequence, genotypes may exhibit similar phenotypes in familiar environments, but may respond differently to novel environmental conditions (Murren et al., 2014; Saltz et al., 2018). Therefore, investigating G × E may help to (1) better understand the vulnerability of populations to climate change, (2) assess the performance of a genotype across environments, and (3) identify traits not having the potential to respond to environmental change (Kelly, 2019; Oostra et al., 2018; Saltz et al., 2018; Sgrò et al., 2016).

To investigate responses to environmental variation, herbivorous insects comprise interesting study systems as they are not only directly but additionally indirectly affected, the latter being mediated by environmentally induced changes in the nutritional quality of their host plants (Harvey et al., 2020). Food quality is an important selective pressure (Chown & Gaston, 2010), and its effects on herbivores have been widely documented (Awmack & Leather, 2002; Scriber & Slansky, 1981). Some recent studies on butterflies, for instance, showed that feeding on plants grown at higher temperatures reduces insect performance, indicated by decreased body size, prolonged development time, and a reduced efficiency of converting food into body matter (Bauerfeind & Fischer, 2013a; Kuczyk, Müller, et al., 2021; Kuczyk, Raharivololoniaina, et al., 2021). With regard to water availability, drought stress may increase plant quality for insects, as evidenced by increased reproductive performance, growth rate, and body size (Franzke & Reinhold, 2011; Kuczyk, Müller, et al., 2021; Larsson, 1989; Salgado & Saastamoinen, 2019). However, to what extent populations from different regions respond variably to changes in plant quality is hitherto largely unexplored.

To address this issue, we here used the temperate zone butterfly *Pieris napi*, which has already served as model organism in various studies on genetic adaptation and phenotypic plasticity. This species seems to possess pronounced plastic and genetic capacities (Espeland et al., 2007; Günter et al., 2019; Günter, Beaulieu, Franke, et al., 2020; Günter, Beaulieu, Freiberg, et al., 2020; Posledovich et al., 2014). For instance, recent studies documented that Italian as compared with German populations seem to have a selective premium on rapid development, being nevertheless larger in size owing to higher larval growth rates (Günter et al., 2019; Günter, Beaulieu, Franke, et al., 2020; Günter, Beaulieu, Freiberg, et al., 2020). Furthermore, feeding on plants grown at cooler temperatures or under drought stress increased the performance of *P*. *napi* (Bauerfeind & Fischer, 2013a; Kuczyk, Müller, et al., 2021). However, whether the effects of variation in host‐plant quality differ across geographic regions, which is the subject of the present study, has not been investigated thus far. To this end, we here used replicated populations from Germany and Italy in a common garden experiment. We manipulated food quality by exposing host plants to different temperature and water regimes, mimicking effects of climate change. We then analyzed selected morphological and physiological traits, indicative of insect performance, in animals fed on plants of the different treatments. We thus exclusively focus on indirect, plant‐mediated effects. We did not additionally investigate direct effects for the following reasons: (1) There are in general many more studies on direct as compared with indirect effects. (2) We have considered both direct and indirect effects in earlier studies on *P*. *napi* already (Bauerfeind & Fischer, 2013a, 2013b). (3) The focus in this study is on variation in the responses of populations from different origins to indirect effects. Additionally addressing direct effects would have been logistically very difficult based on the concomitantly high number of treatments.

Our earlier work has indicated a much faster lifestyle (i.e., a more rapid development) in Italian than in German individuals, arguably to fit in additional generations per year (Günter et al., 2019; Günter, Beaulieu, Freiberg, et al., 2020). This rapid pace of life also seems to increase oxidative damage (Günter, Beaulieu, Franke, et al., 2020). We, therefore, predict that the more time‐stressed Italian populations are more sensitive to poor food quality as compared to their German counterparts, reflecting a cost of rapid development. Thus, we expect G × E with Italian populations responding more strongly to variation in host‐plant quality than German ones.

## 2 MATERIALS AND METHODS

### 2.1 Study organism

*Pieris napi* (Linnaeus, 1758) is a temperate zone butterfly that is widely distributed across northern Eurasia (Ebert & Rennwald, 1991). In most parts of its range, the species has two to three more or less overlapping generations. *P*. *napi* overwinters as pupa (Henriksen & Kreutzer, 1982). The principal larval host plants are several species of the Brassicaceae family, such as *Alliaria petiolata* (Bieb.) and *Cardamine pratensis* (L.). *P*. *napi* is predicted to suffer from climate change due to its association with moist habitats such as moist meadows and forest ecotones (Oliver et al., 2012). In contrast to its relatives *P*. *rapae* and *P*. *brassicae*, *P*. *napi* is of limited importance as a pest species (Ebert & Rennwald, 1991). *P*. *napi* females are polyandrous, that is, they mate repeatedly. Accordingly, males are larger than females due to a positive correlation between body and spermatophore size, with larger spermatophores delaying female remating (Wiklund & Kaitala, 1995).

### 2.2 Population sampling

We collected fecund females from three Italian and three German populations (Figure S1: Appendix S1). In Italy, we sampled 22 females near Mantova (45.21°N, 10.75°E), 20 near Pavia (45.12°N, 9.16°E), and 24 near Torino (45.29°N, 7.30°E). In Germany, we collected 22 females near Rostock (54.11°N, 12.12°E), 25 near Wismar (53.54°N, 11.42°E), and 20 near Greifswald (54.04°N, 13.27°E). Caught females were transferred to Greifswald University and kept in a climate chamber (Sanyo MLR‐351H) at 26°C, 60% relative humidity, and a L18: D6 photoperiod (with light from 5 a.m. to 23 p.m.). Females were placed individually into small plastic pots (1 L) covered with gauze and were provided with *Alliaria petiolata* as oviposition substrate, and additionally fresh flowers, water, and a highly concentrated sucrose solution (ca. 20 vol%) for adult feeding. Deposited eggs were collected daily and transferred to small plastic boxes.

### 2.3 Host‐plant treatments

To investigate the effects of host‐plant manipulation on *P*. *napi* performance, we used *Sinapis alba* L. (Brassicaceae), a widespread annual plant of the temperate zone with a short life cycle (Dong et al., 2012). It is a common crop plant but also grows on ruderal places. Plants were grown from seeds (Rühlemann's, Horstedt, Germany) in commercially available potting soil in plastic pots (11 × 11 × 12 cm; 5–6 plants per pot) kept in climate cabinets (Sanyo MLR‐351H) at 70% relative humidity and a L18: D6 photoperiod. Seedlings were divided among four treatments once the first leaves following the cotyledons had appeared. We used 2 temperature and 2 water regimes with 10 plant pots per treatment: (1) 17°C, water control (150 ml of water per day and pot); (2) 17°C, drought (50 ml water per day); (3) 24°C, water control; (4) 24°C, drought. The water control treatment was chosen to keep plants fully turgid until the next watering bout, based on pre‐trials. Leaves were harvested for larval feeding after plants had reached the eight‐leaf stage. The above plant treatments have been shown to affect *S*. *alba* chemistry by increasing leaf glucosinolate and decreasing C:N ratio at higher temperatures (Kuczyk, Müller, et al., 2021).

### 2.4 Insect rearing and analyses

After hatching, F1 larvae were randomly divided among the four host‐plant treatments, using 100 individuals per population and treatment (i.e., 2400 in total, 6 populations × 4 treatments × 100). Five larvae each were reared within small plastic boxes (125 ml) in a climate chamber (Sanyo MLR‐351H) under the same conditions as used for oviposition (26°C, 60% relative humidity, L18:D6 photoperiod). Larvae were supplied daily with fresh leaves from the respective host‐plant treatment ad libitum. Boxes were checked daily and larvae were supplied with fresh leaves as necessary. Resulting pupae were weighted one day after pupation (Sartorius CPA225D) and afterwards kept individually until eclosion. Development time was scored from hatching until adult eclosion, and larval growth rate was calculated as mass gain per day (pupal mass divided by larval development time). After eclosion, adult butterflies were frozen at −80°C until further analyses.

We measured, in addition, the following morphological and physiological parameters as indicators of butterfly performance: total body mass, thorax mass, abdomen mass, thorax‐abdomen ratio (as proxies of body size and the relative investment into flight versus reproduction), forewing length, wing loading and wing aspect ratio (as measures of flight performance), abdomen fat content, and phenoloxidase (PO) activity (reflecting general condition in insects; Freitak et al., 2003; González‐Santoyo & Córdoba‐Aguilar, 2011). Thus, we considered an array of traits as fitness proxies, including development time and growth rate, body size, flight performance, and condition, which may all be well relevant for survivorship and reproduction. First, total body mass was determined to the nearest 0.01 mg. Then, wings, head, and legs were removed. Thorax and abdomen were separated and afterward weighed. Forewing length and area were measured using digital images of left forewings. Images were captured ventrally with a digital camera mounted on a stereo microscope. Wing loading was calculated as total body mass divided by forewing area and wing aspect ratio as 4 × forewing length^2^ divided by forewing area (Berwaerts et al., 2002).

Abdomen fat content was measured according to Fischer et al. (2003) but using acetone instead of dichloromethane. In short, abdomens were dried to constant weight for 2 days at 70°C (UN110, Memmert) and initial dry mass was taken. Thereafter, fat was extracted twice (2 × 2 days) using 1.5 ml of acetone for each butterfly. Then, abdomens were dried again for 2 days and the fat‐free dry mass was measured. Relative fat content was determined as the mass difference between initial and final abdomen dry mass and is given in percent. For PO measurements, thoraces were homogenized (Tissuelyser II, Qiagen) with 200 µl of a phosphate‐buffered saline (PBS) and centrifuged for 10 min at 14,000 rpm. Afterward, 60 µl of the supernatant or PBS buffer (for blank measurements) was transferred to a microtiter plate (96 wells) and 140 µl l‐Dopa (dihydrophenyl‐l‐alanine; 10 mM in PBS buffer) was added. Readings were taken every 30 s on a spectrophotometric plate reader (BioTek EL 808) at 30°C and 490 nm for 45 min. Enzyme activity was measured as the slope during the linear phase of the reaction, during which the enzyme catalyzes the transition from l‐Dopa to dopachrome. PO activity was assayed twice per individual. The mean of both readings, corrected for blank values, was used for statistical analyses. Total protein content was quantified using the Roti Nanoquant protein assay based on the Bradford method (Bradford, 1976), following the manufacturer's instructions (Roth).

### 2.5 Statistical analyses

All traits were analyzed with general linear mixed models (GLMMs) using population origin, host‐plant temperature, host‐plant water treatment, butterfly sex, and all interactions as fixed factors. Population (nested within origin) was included as a random effect. Protein was added as a covariate in the analysis of PO activity. Minimum adequate models were built by sequentially removing non‐significant interaction terms. Pupal mass was ln‐transformed prior to analyses to meet GLMM requirements. Mean values depicted in the tables and figures refer to untransformed data. All means are given ± 1 SE. All statistical tests were performed using Statistica 8.0 (StatSoft, 2007).

## 3 RESULTS

Origin significantly affected fat content only, while replicate populations differed significantly in all traits (Table 1). Fat content was higher in German than in Italian individuals (G: 11.9 ± 0.2% > I: 4.9 ± 0.2%). However, origin was involved in interactions with three other factors, indicating that animals from different origins showed differential responses to environmental variation or among sexes (see further below).

**TABLE 1:** Results of general linear mixed models for the effects of population origin, host‐plant temperature, host‐plant water regime, and sex (all fixed) on various traits in *Pieris napi*

	MS	df	*F*	*p*
Development time				
Origin	4080.8	1	3.13	.1518
Population [Origin]	1310.9	4	675.31	**<.0001**
Temperature	18.3	1	9.43	.**0022**
Water	2.3	1	1.18	.2782
Sex	29.3	1	15.10	.**0001**
Origin × Temp.	8.7	1	4.50	.**0342**
Origin × Water	13.8	1	7.11	.**0078**
Error	1.9	1003		
Larval growth rate				
Origin	2.62	1	2.58	.1833
Population [Origin]	1.02	4	473.94	**<.0001**
Temperature	0.02	1	7.96	.**0049**
Water	<0.01	1	0.03	.8710
Sex	0.07	1	33.24	**<.0001**
Origin × Temp.	0.02	1	9.34	.**0023**
Origin × Water	0.02	1	10.16	.**0015**
Error	<0.01	1002		
Pupal mass				
Origin	4.52	1	3.63	.1296
Population [Origin]	1.25	4	54.27	**<.0001**
Temperature	0.17	1	7.50	.**0063**
Water	0.06	1	2.79	.0949
Sex	3.30	1	143.02	**<.0001**
Origin × Temp.	0.29	1	12.47	.**0004**
Error	0.02	1003		
Thorax mass				
Origin	2703.5	1	5.11	.0865
Population [Origin]	530.8	4	58.01	**<.0001**
Temperature	108.5	1	11.85	.**0006**
Water	8.1	1	0.88	.3477
Sex	1107.5	1	121.03	**<.0001**
Origin × Temp.	72.2	1	7.89	.**0051**
Error	9.2	1004		
Abdomen mass				
Origin	5701.8	1	3.93	.1186
Population [Origin]	1458.4	4	52.63	**<.0001**
Temperature	115.9	1	4.18	.**0411**
Water	2.9	1	0.11	.7449
Sex	178.5	1	6.44	.**0113**
Error	27.7	1005		
Thorax‐abdomen ratio				
Origin	1.03	1	3.69	.1270
Population [Origin]	0.28	4	7.92	**<.0001**
Temperature	0.02	1	0.45	.5004
Water	0.04	1	1.10	.2948
Sex	2.28	1	64.65	**<.0001**
Temp. × Water	0.31	1	8.67	.**0033**
Error	0.04	1004		
Forewing length				
Origin	109.6	1	1.86	.2442
Population [Origin]	64.5	1	26.64	**<.0001**
Temperature	3.7	4	1.54	.2141
Water	8.5	1	3.50	.0615
Sex	2.4	1	0.97	.3246
Origin × Temp.	16.4	1	6.79	.**0093**
Origin × Sex	16.8	1	6.96	.**0085**
Error	2.4	1320		
Wing loading				
Origin	0.017	1	0.07	.8028
Population [Origin]	0.268	4	110.26	**<.0001**
Temperature	0.004	1	1.67	.1962
Water	0.002	1	0.70	.4027
Sex	0.004	1	1.80	.1796
Error	0.002	1320		
Wing aspect ratio				
Origin	3.49	1	3.44	.1348
Population [Origin]	1.09	4	5.26	.**0003**
Temperature	0.04	1	0.21	.6448
Water	0.44	1	2.13	.1450
Sex	3.27	1	15.80	**<.0001**
Origin × Sex	2.34	1	11.34	.**0008**
Error	0.21	1319		
Fat content %				
Origin	12293.5	1	18.77	.**0123**
Population [Origin]	657.7	4	24.70	**<.0001**
Temperature	251.2	1	9.43	.**0022**
Water	6.7	1	0.25	.6173
Sex	50.2	1	1.89	.1700
Error	26.6	1003		
Phenoloxidase activity				
Origin	188.7	1	0.58	.4869
Population [Origin]	402.0	4	16.37	**<.0001**
Temperature	182.6	1	7.44	.**0065**
Water	10.3	1	0.42	.5183
Sex	69.6	1	2.83	.0926
Protein	785.6	1	32.00	**<.0001**
Error	24.6	1004		

Note

Replicate population was added as a random factor and nested within origin. Protein content was added as a covariate in the analysis of phenoloxidase activity. Models were constructed by sequentially removing nonsignificant interaction terms. Significant *p*‐values are given in bold. Development time = larval time + pupal time.

Host‐plant water treatment did not significantly affect any butterfly trait directly. However, host‐plant temperature caused significant variation in development time, larval growth rate, pupal mass, thorax mass, abdomen mass, fat content, and PO activity (Table 1). Feeding on plants grown at the higher compared with the lower temperature decreased growth rates (17°C: 0.389 ± 0.002 mg/day > 24°C: 0.380 ± 0.002 mg/day), pupal mass (17°C: 107.7 ± 0.7 mg > 24°C: 105.2 ± 0.7 mg), thorax mass (17°C: 14.3 ± 0.1 mg > 24°C: 13.7 ± 0.1 mg), abdomen mass (17°C: 16.3 ± 0.2 mg > 24°C:15.6 ± 0.2 mg), fat content (17°C: 8.9 ± 0.2% > 24°C: 7.9 ± 0.2%), and PO activity (17°C: 5.0 ± 0.2 mOD/ml > 24°C: 4.1 ± 0.2 mOD/ml) but increased development time (17°C: 20.03 ± 0.06 days < 24°C: 20.30 ± 0.06 days).

Butterfly sex significantly influenced development time, larval growth rate, pupal mass, thorax mass, abdomen mass, thorax‐abdomen ratio, and wing aspect ratio (Table 1). Males compared to females had higher larval growth rates (♂: 0.393 ± 0.002 mg/day > ♀: 0.376 ± 0.002 mg/day), pupal masses (♂: 112.5 ± 0.7 mg > ♀: 100.4 ± 0.7 mg), thorax masses (♂: 15.0 ± 0.1 mg > ♀: 12.9 ± 0.1 mg), abdomen masses (♂: 16.4 ± 0.2 mg > ♀: 15.5 ± 0.2 mg), thorax‐abdomen ratios (♂: 0.97 ± 0.01 > ♀: 0.88 ± 0.01), wing aspect ratios (♂: 10.44 ± 0.02 > ♀: 10.33 ± 0.02), and a shorter larval development time (♂:19.99 ± 0.06 days < ♀: 20.33 ± 0.06 days).

**FIGURE 1:**
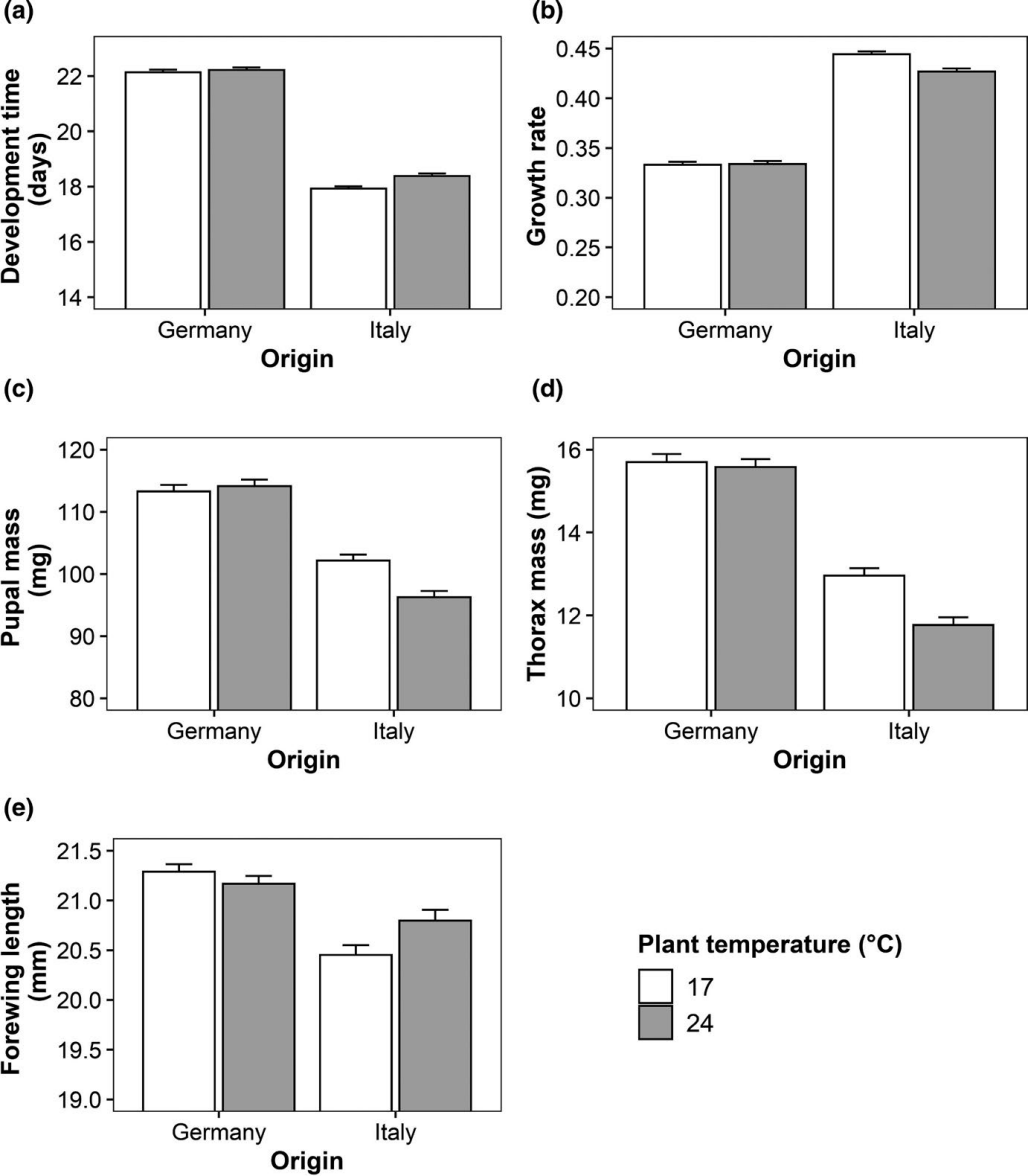
Effects of origin (Germany vs. Italy) and host‐plant temperature (17 vs. 24°C) on development time (a), larval growth rate (b), pupal mass (c), thorax mass (d), and forewing length (e) in *Pieris napi*. Given are means + SE

**FIGURE 2:**
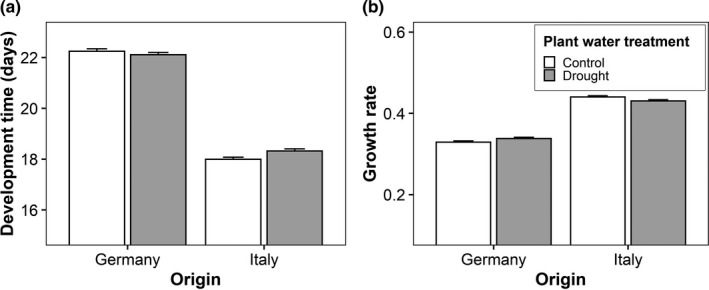
Effects of origin (Germany vs. Italy) and host‐plant water treatment (control vs. drought) on development time (a) and larval growth rate (b) in *Pieris napi*. Given are means + SE

**FIGURE 3:**
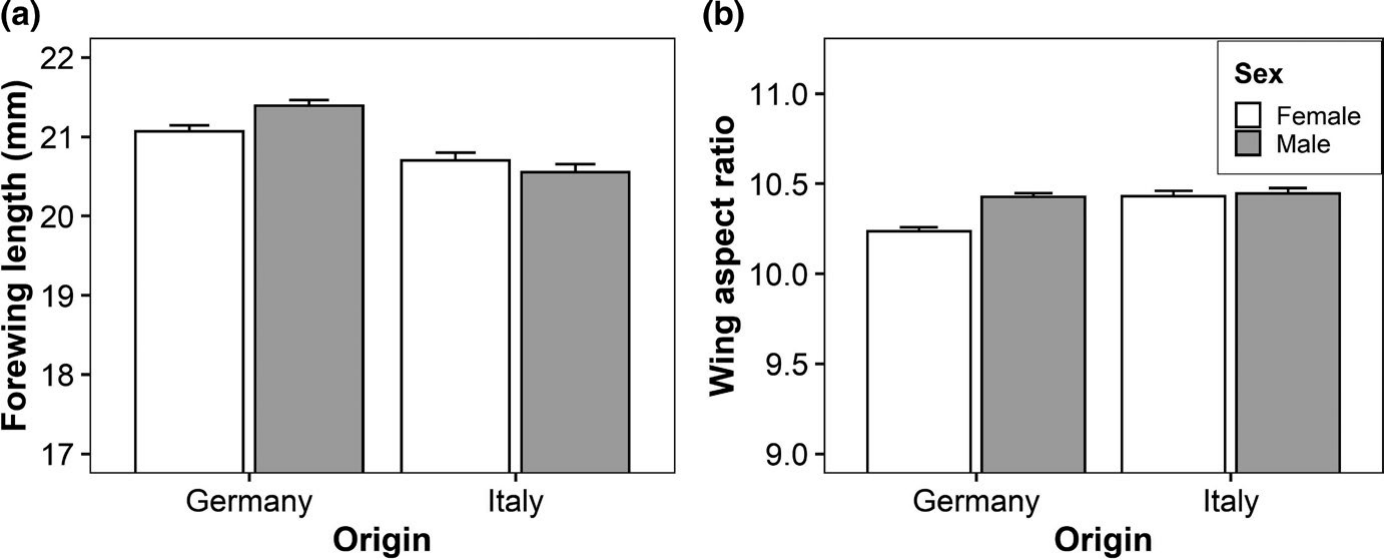
Effects of origin (Germany vs. Italy) and butterfly sex on forewing length (a) and wing aspect ratio (b) in *Pieris napi*. Given are means + SE

Significant interactions between butterfly origin and host‐plant temperature were found for development time, larval growth rate, pupal mass, thorax mass, and forewing length (Table 1). These indicate that the effects of host‐plant temperature were restricted to Italian individuals while German ones remained unaffected. Feeding on plants grown at the higher temperature decreased larval growth rate, pupal mass, and thorax mass but increased development time and wing length in the Italian individuals (Figure 1). The interaction between butterfly origin and plant water treatment was significant for development time and larval growth rate (Table 1). Similar to above, effects of the host‐plant water treatment were found in Italian individuals only. They showed longer development times and lower larval growth rates when feeding on drought‐stressed plants compared with control plants (Figure 2). The interaction between origin and sex was significant for forewing length and wing aspect ratio (Table 1). For both traits, there was no significant difference between the sexes in Italian individuals, while German males had longer wings and higher wing aspect ratios than German females (Figure 3). Additionally, the interaction between host‐plant temperature and water treatment significantly affected the thorax‐abdomen ratio (Table 1). Feeding on plants from the drought treatment increased the thorax‐abdomen ratio when in combination with the low temperature treatment (17°C drought: 0.95 ± 0.01; 17°C control: 0.91 ± 0.01), but tended to decrease it when in combination with the high temperature treatment (24°C drought: 0.91 ± 0.01; 24°C control: 0.93 ± 0.01).

## 4 DISCUSSION

Organisms are increasingly confronted with environmental variability and extremes owing to anthropogenic climate change (Harvey et al., 2020; Soroye et al., 2020). Phenotypic plasticity is a potentially powerful mechanism to cope with such variability. Here, we discuss patterns of geographic variation in phenotypic plasticity in an insect herbivore in response to changes in host‐plant quality, and its potential significance for dealing with climate change.

### 4.1 Effects of geographic origin

With regard to the main effects of geographic origin, a significant difference was found only for fat content. The higher fat content found in German butterflies fits the previously observed differences in lifestyles (Günter, Beaulieu, Franke, et al., 2020; Günter, Beaulieu, Freiberg, et al., 2020). It may thus indicate that storage reserves are more strongly reduced in the rapidly developing Italian individuals compared with German individuals. However, we could not confirm the earlier results on differences in development time, growth rates, and body size among Italian and German populations (Günter, Beaulieu, Franke, et al., 2020; Günter, Beaulieu, Freiberg, et al., 2020). This is, at least in part, likely due to the pronounced variation among populations within countries (see Figure S2: Appendix S1 for further illustration), in combination with the low statistical power of nested GLMs when the number of replicates is low. Note the substantial phenotypic difference in development time and growth rate between German and Italian populations, in accordance with previous results, also in the present study (Figure 1).

Indeed, populations from Germany and Italy differed in their responses to host‐plant quality, as indicated by significant G × E suggesting genetic variation in phenotypic plasticity (Via & Lande, 1985). Such interactions occurred most frequently between origin and host‐plant temperature (five significant interactions). Specifically, feeding on plants grown at the higher temperature diminished performance of Italian individuals by decreasing larval growth rate, pupal mass, and thorax mass but increasing development time, while German individuals remained unaffected (Figure 1). Furthermore, two significant interactions between origin and plant water treatment were found, again showing that the plant treatment only affected Italian individuals. These showed longer development times and lower larval growth rates when feeding on drought‐stressed compared with control plants (Figure 2). The above findings indicate, as predicted, that Italian populations (1) respond more strongly to variation in host‐plant quality and (2) are more sensitive to poor food quality than German ones. This may, consequently, reflect a cost of the rapid lifestyle of Italian individuals (Günter, Beaulieu, Franke, et al., 2020; Günter, Beaulieu, Freiberg, et al., 2020). The fact that wing length actually increased when feeding on plants grown at the higher temperature, despite reduced pupal and thorax mass, may seem contradictory upon first sight. This is because wing length is typically positively related to body mass. However, we suggest that it may reflect increased investment into dispersal ability to escape areas with adverse, that is, very warm, environmental conditions (Matthysen, 2012), in which plants are of reduced quality.

### 4.2 Effects of host‐plant treatment

Overall, our results clearly indicate detrimental effects of high plant‐growing temperature on the performance of *P*. *napi*. This is evidenced by a prolonged development time and reduced larval growth rate, body mass, fat content, and PO activity when having fed on plants grown at the higher rather than lower temperature. These findings are in line with other studies on insects (Bauerfeind & Fischer, 2013a; Kuczyk, Müller, et al., 2021; Kuczyk, Raharivololoniaina, et al., 2021; but Raharivololoniaina et al., 2021). The specific mechanisms underlying the reduced performance are unclear, but temperature‐mediated changes in leaf glucosinolate, carbon, and carbon:nitrogen ratio, as have been documented in *S*. *alba*, may play a significant role (Kuczyk, Müller, et al., 2021). However, plant water regime did not affect any trait directly, and was involved in two interactions only. Thus, we could not confirm that drought stress increases food quality for herbivores in line with the plant–stress hypothesis (Franzke & Reinhold, 2011; Kuczyk, Müller, et al., 2021; Larsson, 1989; Salgado & Saastamoinen, 2019). Note that we have even found some weak evidence for negative effects of drought stress, as development times were longer and larval growth rates reduced when feeding on drought‐stressed compared with control plants (Figure 2). We have no explanation for this deviation, but the results may depend on the specific treatments applied. Thus, we speculate that the severity of drought stress and interactions with temperature, with high temperatures increasing drought stress, may play a role (Kuczyk, Müller, et al., 2021). Assay conditions are well known to affect the outcome of biological experiments (e.g., Fischer et al., 2011; Petavy et al., 2001).

### 4.3 Sexual differences

We found several sex differences that are typical for *P*. *napi* (Günter, Beaulieu, Freiberg, et al., 2020; Kuczyk, Müller, et al., 2021). In particular, males showed protandry, that is, they eclosed before females, to maximize mating opportunity (Fagerström & Wiklund, 1982; Wiklund & Fagerstrom, 1977), which was facilitated by higher larval growth rates. Males were larger than females, which is quite exceptional for an insect, but in the case of *P*. *napi*, this is driven by a covariance between body and spermatophore size. As males of this species are polyandrous, transferring a larger spermatophore ensures paternity by delaying remating (Wiklund & Kaitala, 1995). The lower thorax‐abdomen ratio found in females likely reflects increased investment into the abdomen and thus fecundity selection, while males invest relatively more into flight muscles (Berwaerts et al., 2002; Honěk, 1993). A higher wing aspect ratio, finally, has been repeatedly found in male butterflies. It may increase flight ability, in particular maneuverability, which might be beneficial during mate location and courtship (Berwaerts et al., 2002, 2006).

## 5 CONCLUSIONS

In general, *P*. *napi* exhibits pronounced genetic and plastic variation, which may indicate high adaptive capacities (Günter, Beaulieu, Freiberg, et al., 2020). While previous studies on this species have assessed exclusively direct (e.g., Bauerfeind & Fischer, 2013c; Bauerfeind & Fischer, 2014) or both direct and indirect effects (Bauerfeind & Fischer, 2013a, 2013b), we here explicitly focused on variation in response to indirect effects across populations. Our recent findings suggest that Italian populations respond more strongly to variation in host‐plant quality and are more sensitive to poor food quality than German ones. In terms of vulnerability to climate change, two conclusions seem important. First, German individuals appear to respond less plastically to variation in food quality. This, however, does not seem to be disadvantageous facing climate change. Rather, it seems that these slow developing populations are more resilient against environmental perturbations (at least with regard to food quality), and may therefore potentially even benefit from warmer temperatures, perhaps allowing additional generations per year. Second, Italian individuals with their rapid lifestyle are probably not able to benefit from warmer conditions which reduce development time, as they will likely suffer from the concomitantly reduced host‐plant quality. These “warm‐adapted” populations appear, therefore, to be more at risk from climate change than their northern counterparts, potentially reflecting a cost of their rapid lifestyle (Günter, Beaulieu, Freiberg, et al., 2020; Günter, Beaulieu, Freiberg, et al., 2020). We believe that our results have wide‐ranging implications also for other herbivorous insects, as it seems likely that climate change will have pronounced effects on plant chemistry (DeLucia et al., 2012; Kuczyk, Müller, et al., 2021; Kuczyk, Raharivololoniaina, et al., 2021; Suseela & Tharayil, 2018). It seems also conceivable that time‐stressed species or populations respond generally more strongly to poor food quality, based on a high demand for nutrients to fuel rapid development. However, since we did not consider the combination of both direct and indirect effects, which may both be of relevance in nature, our results should be interpreted with caution. Nevertheless, our study exemplifies how investigating G × E may help to better understand the vulnerability of populations to climate change.

## CONFLICT OF INTEREST

None declared.

## AUTHOR CONTRIBUTIONS

**Josephine Kuczyk:** Data curation (equal); Formal analysis (equal); Investigation (lead); Validation (equal); Visualization (equal); Writing‐original draft (equal); Writing‐review & editing (equal). **Ange Raharivololoniaina:** Formal analysis (equal); Validation (equal); Visualization (equal); Writing‐original draft (equal); Writing‐review & editing (equal). **Klaus Fischer:** Conceptualization (equal); Data curation (equal); Formal analysis (equal); Funding acquisition (lead); Investigation (supporting); Methodology (equal); Project administration (equal); Resources (lead); Supervision (lead); Validation (equal); Writing‐original draft (supporting); Writing‐review & editing (equal).

## Supporting information

Appendix S1

## Data Availability

Data from this study are available from the Dryad Digital Repository: https://doi.org/10.5061/dryad.g1jwstqs1.

## References

[ece38392-bib-0001] Awmack, C. S. , & Leather, S. R. (2002). Host plant quality and fecundity in herbivorous insects. Annual Review of Entomology, 47, 817–844. 10.1146/annurev.ento.47.091201.145300 11729092

[ece38392-bib-0002] Bauerfeind, S. S. , & Fischer, K. (2013a). Increased temperature reduces herbivore host‐plant quality. Global Change Biology, 19, 3272–3282. 10.1111/gcb.12297 23775632

[ece38392-bib-0003] Bauerfeind, S. S. , & Fischer, K. (2013b). Testing the plant stress hypothesis: Stressed plants offer better food to an insect herbivore. Entomologia Experimentalis et Applicata, 149, 148–158. 10.1111/eea.12118

[ece38392-bib-0004] Bauerfeind, S. S. , & Fischer, K. (2013c). Targeting the right trait: The relative suitability of a host plant depends on the herbivore trait considered and ambient temperature. Basic and Applied Ecology, 14, 555–564. 10.1016/j.baae.2013.08.010

[ece38392-bib-0005] Bauerfeind, S. S. , & Fischer, K. (2014). Integrating temperature and nutrition – environmental impacts on an insect immune system. Journal of Insect Physiology, 64, 14–20. 10.1016/j.jinsphys.2014.03.003 24636910

[ece38392-bib-0006] Berwaerts, K. , Aerts, P. , & van Dyck, H. (2006). On the sex‐specific mechanisms of butterfly flight: Flight performance relative to flight morphology, wing kinematics, and sex in *Pararge* *aegeria* . Biological Journal of the Linnean Society, 89, 675–687. 10.1111/j.1095-8312.2006.00699.x

[ece38392-bib-0007] Berwaerts, K. , Van Dyck, H. , & Aerts, P. (2002). Does flight morphology relate to flight performance? An experimental test with the butterfly *Pararge* *aegeria* . Functional Ecology, 16, 484–491.

[ece38392-bib-0008] Blanckenhorn, W. U. , & Demont, M. (2004). Bergmann and converse Bergmann latitudinal clines in arthropods: Two ends of a continuum? Integrative and Comparative Biology, 44, 413–424. 10.1093/icb/44.6.413 21676727

[ece38392-bib-0009] Bradford, M. M. (1976). A rapid and sensitive method for the quantitation of microgram quantities of protein utilizing the principle of protein‐dye binding. Analytical Biochemistry, 72, 248–254. 10.1016/0003-2697(76)90527-3 942051

[ece38392-bib-0010] Chown, S. L. , & Gaston, K. J. (2010). Body size variation in insects: A macroecological perspective. Biological Reviews, 85, 139–169. 10.1111/j.1469-185X.2009.00097.x 20015316

[ece38392-bib-0011] DeLucia, E. H. , Nabity, P. D. , Zavala, J. A. , & Berenbaum, M. R. (2012). Climate change: Resetting plant‐insect interactions. Plant Physiology, 160, 1677–1685. 10.1104/pp.112.204750 22972704 PMC3510101

[ece38392-bib-0012] Dong, C.‐H. , Li, C. , Yan, X.‐H. , Huang, S.‐M. , Huang, J.‐Y. , Wang, L.‐J. , Guo, R.‐X. , Lu, G.‐Y. , Zhang, X.‐K. , Fang, X.‐P. , & Wei, W.‐H. (2012). Gene expression profiling of *Sinapis* *alba* leaves under drought stress and rewatering growth conditions with Illumina deep sequencing. Molecular Biology Reports, 39, 5851–5857. 10.1007/s11033-011-1395-9 22207172

[ece38392-bib-0013] Ebert, G. , & Rennwald, E. (1991). Die Schmetterlinge Baden‐Württembergs. Ulmer.

[ece38392-bib-0014] Espeland, M. , Aagaard, K. , Balstad, T. , & Hindar, K. (2007). Ecomorphological and genetic divergence between lowland and montane forms of the *Pieris* *napi* species complex (Pieridae, Lepidoptera). Biological Journal of the Linnean Society, 92, 727–745.

[ece38392-bib-0015] Fagerström, T. , & Wiklund, C. (1982). Why do males emerge before females? Protandry as a mating strategy in male and female butterflies. Oecologia, 52, 164–166. 10.1007/BF00363830 28310501

[ece38392-bib-0016] Fischer, K. , Brakefield, P. M. , & Zwaan, B. J. (2003). Plasticity in butterfly egg size: Why larger offspring at lower temperatures? Ecology, 84, 3138–3147. 10.1890/02-0733

[ece38392-bib-0017] Fischer, K. , & Karl, I. (2010). Exploring plastic and genetic responses to temperature variation using copper butterflies. Climate Research, 43, 17–30. 10.3354/cr00892

[ece38392-bib-0018] Fischer, K. , Kölzow, N. , Höltje, H. , & Karl, I. (2011). Assay conditions in laboratory experiments: Is the use of constant rather than fluctuating temperatures justified when investigating temperature‐induced plasticity? Oecologia, 166, 23–33. 10.1007/s00442-011-1917-0 21286923

[ece38392-bib-0019] Franke, K. , Karl, I. , Centeno, T. P. , Feldmeyer, B. , Lassek, C. , Oostra, V. , Riedel, K. , Stanke, M. , Wheat, C. W. , & Fischer, K. (2019). Effects of adult temperature on gene expression in a butterfly: Identifying pathways associated with thermal acclimation. BMC Evolutionary Biology, 19, 1–16. 10.1186/s12862-019-1362-y 30674272 PMC6345059

[ece38392-bib-0020] Franzke, A. , & Reinhold, K. (2011). Stressing food plants by altering water availability affects grasshopper performance. Ecosphere, 2, 1–13. 10.1890/ES11-00095.1

[ece38392-bib-0021] Freitak, D. , Ots, I. , Vanatoa, A. , & Hõrak, P. (2003). Immune response is energetically costly in white cabbage butterfly pupae. Proceedings of the Royal Society of London. Series B: Biological Sciences, 270, S220–S222. 10.1098/rsbl.2003.0069 PMC1809938 14667388

[ece38392-bib-0022] González‐Santoyo, I. , & Córdoba‐Aguilar, A. (2011). Phenoloxidase: A key component of the insect immune system. Entomologia Experimentalis et Applicata, 142, 1–16.

[ece38392-bib-0023] Günter, F. , Beaulieu, M. , Brunetti, M. , Lange, L. , Schmitz Ornés, A. , & Fischer, K. (2019). Latitudinal and altitudinal variation in ecologically important traits in a widespread butterfly. Biological Journal of the Linnean Society, 128, 742–755. 10.1093/biolinnean/blz133

[ece38392-bib-0024] Günter, F. , Beaulieu, M. , Franke, K. , Toshkova, N. , & Fischer, K. (2020). Clinal variation in investment into reproduction versus maintenance suggests a ‘pace‐of‐life’ syndrome in a widespread butterfly. Oecologia, 193, 1011–1020. 10.1007/s00442-020-04719-4 32719946 PMC7458933

[ece38392-bib-0025] Günter, F. , Beaulieu, M. , Freiberg, K. F. , Welzel, I. , Toshkova, N. , Žagar, A. , Simčič, T. , & Fischer, K. (2020). Genotype‐environment interactions rule the response of a widespread butterfly to temperature variation. Journal of Evolutionary Biology, 33, 920–929. 10.1111/jeb.13623 32243031

[ece38392-bib-0026] Hansen, J. , Sato, M. , & Ruedy, R. (2012). Perception of climate change. Proceedings of the National Academy of Sciences of the United States of America, 109, E2415–E2423. 10.1073/pnas.1205276109 22869707 PMC3443154

[ece38392-bib-0027] Harvey, J. A. , Heinen, R. , Gols, R. , & Thakur, M. P. (2020). Climate change‐mediated temperature extremes and insects: From outbreaks to breakdowns. Global Change Biology, 26, 6685–6701. 10.1111/gcb.15377 33006246 PMC7756417

[ece38392-bib-0028] Henriksen, H. J. , & Kreutzer, I. (1982). The butterflies of Scandinavia in nature. Skandinavisk Bogforlag.

[ece38392-bib-0029] Honěk, A. , & Honek, A. (1993). Intraspecific variation in body size and fecundity in insects: A general relationship. Oikos, 66, 483–492. 10.2307/3544943

[ece38392-bib-0030] Kawecki, T. J. , & Ebert, D. (2004). Conceptual issues in local adaptation. Ecology Letters, 7, 1225–1241. 10.1111/j.1461-0248.2004.00684.x

[ece38392-bib-0031] Kelly, M. (2019). Adaptation to climate change through genetic accommodation and assimilation of plastic phenotypes. Philosophical Transactions of the Royal Society B: Biological Sciences, 374, 1–10. 10.1098/rstb.2018.0176 PMC6365860 30966963

[ece38392-bib-0032] Kuczyk, J. , Müller, C. , & Fischer, K. (2021). Plant‐mediated indirect effects of climate change on an insect herbivore. Basic and Applied Ecology, 53, 100–113. 10.1016/j.baae.2021.03.009

[ece38392-bib-0033] Kuczyk, J. , Raharivololoniaina, A. , & Fischer, K. (2021). High temperature and soil moisture reduce host‐plant quality for an insect herbivore. Ecological Entomology, 46(4), 889–897. 10.1111/een.13025

[ece38392-bib-0034] Larsson, S. (1989). Stressful times for the plant stress – Insect performance hypothesis. Oikos, 56, 277–283. 10.2307/3565348

[ece38392-bib-0035] Matthysen, E. (2012). Multicausality of dispersal: A review. Dispersal Ecology and Evolution, 27, 3–18.

[ece38392-bib-0036] Murren, C. J. , Maclean, H. J. , Diamond, S. E. , Steiner, U. K. , Heskel, M. A. , Handelsman, C. A. , Ghalambor, C. K. , Auld, J. R. , Callahan, H. S. , Pfennig, D. W. , Relyea, R. A. , Schlichting, C. D. , & Kingsolver, J. (2014). Evolutionary change in continuous reaction norms. The American Naturalist, 183, 453–467. 10.1086/675302 24642491

[ece38392-bib-0037] Nygren, G. H. , Bergström, A. , & Nylin, S. (2008). Latitudinal body size clines in the butterfly *Polyommatus* *icarus* are shaped by gene‐environment interactions. Journal of Insect Science, 8, 1–13.

[ece38392-bib-0038] Oliver, T. H. , Thomas, C. D. , Hill, J. K. , Brereton, T. , & Roy, D. B. (2012). Habitat associations of thermophilous butterflies are reduced despite climatic warming. Global Change Biology, 18, 2720–2729. 10.1111/j.1365-2486.2012.02737.x 24501051

[ece38392-bib-0039] Oomen, R. A. , & Hutchings, J. A. (2015). Genetic variability in reaction norms in fishes. Environmental Reviews, 23, 353–366. 10.1139/er-2014-0077

[ece38392-bib-0040] Oostra, V. , Saastamoinen, M. , Zwaan, B. J. , & Wheat, C. W. (2018). Strong phenotypic plasticity limits potential for evolutionary responses to climate change. Nature Communications, 9, 1–11. 10.1038/s41467-018-03384-9 PMC5843647 29520061

[ece38392-bib-0041] Petavy, G. , David, J. R. , Gibert, P. , & Moreteau, B. (2001). Viability and rate of development at different temperatures in *Drosophila*: A comparison of constant and alternating thermal regimes. Journal of Thermal Biology, 26, 29–39. 10.1016/S0306-4565(00)00022-X 11070342

[ece38392-bib-0042] Posledovich, D. , Toftegaard, T. , Navarro‐Cano, J. A. , Wiklund, C. , Ehrlén, J. , & Gotthard, K. (2014). Latitudinal variation in thermal reaction norms of post‐winter pupal development in two butterflies differing in phenological specialization. Biological Journal of the Linnean Society, 113, 981–991. 10.1111/bij.12371

[ece38392-bib-0043] Raharivololoniaina, A. , Berweiler, S. , & Fischer, K. (2021). Nitrogen fertilization and high plant growing temperature increase herbivore performance. Ecosphere.

[ece38392-bib-0044] Rahmstorf, S. , & Coumou, D. (2011). Increase of extreme events in a warming world. Proceedings of the National Academy of Sciences of the United States of America, 108, 17905–17909. 10.1073/pnas.1101766108 22025683 PMC3207670

[ece38392-bib-0045] Rodrigues, Y. K. , & Beldade, P. (2020). Thermal plasticity in insects’ response to climate change and to multifactorial environments. Frontiers in Ecology and Evolution, 8, 1–12. 10.3389/fevo.2020.00271

[ece38392-bib-0046] Salgado, A. L. , & Saastamoinen, M. (2019). Developmental stage‐dependent response and preference for host plant quality in an insect herbivore. Animal Behaviour, 150, 27–38. 10.1016/j.anbehav.2019.01.018 31024189 PMC6467838

[ece38392-bib-0047] Saltz, J. B. , Bell, A. M. , Flint, J. , Gomulkiewicz, R. , Hughes, K. A. , & Keagy, J. (2018). Why does the magnitude of genotype‐by‐environment interaction vary? Ecology and Evolution, 8, 6342–6353. 10.1002/ece3.4128 29988442 PMC6024136

[ece38392-bib-0048] Sambucetti, P. , Loeschcke, V. , & Norry, F. M. (2006). Developmental time and size‐related traits in *Drosophila* *buzzatii* along an altitudinal gradient from Argentina. Hereditas, 143, 77–83. 10.1111/j.2006.0018-0661.01934.x 17362338

[ece38392-bib-0049] Scriber, J. M. , & Slansky, F. (1981). The nutritional ecology of immature insects. Annual Review of Entomology, 26, 183–211. 10.1146/annurev.en.26.010181.001151

[ece38392-bib-0050] Sgrò, C. M. , Terblanche, J. S. , & Hoffmann, A. A. (2016). What can plasticity contribute to insect responses to climate change? Annual Review of Entomology, 61, 433–451. 10.1146/annurev-ento-010715-023859 26667379

[ece38392-bib-0051] Soroye, P. , Newbold, T. , & Kerr, J. T. (2020). Climate change contributes to widespread declines among bumble bees across continents. Science, 367, 685–688. 10.1126/science.aax8591 32029628

[ece38392-bib-0052] Suseela, V. , & Tharayil, N. (2018). Decoupling the direct and indirect effects of climate on plant litter decomposition: Accounting for stress‐induced modifications in plant chemistry. Global Change Biology, 24, 1428–1451. 10.1111/gcb.13923 28986956

[ece38392-bib-0053] Via, S. , & Lande, R. (1985). Genotype‐environment interaction and the evolution of phenotypic plasticity. Evolution, 39, 505–522. 10.1111/j.1558-5646.1985.tb00391.x 28561964

[ece38392-bib-0054] Wiklund, C. , & Fagerstrom, T. (1977). Why do males emerge before females? Oecologia, 31, 153–158. 10.1007/BF00346917 28309135

[ece38392-bib-0055] Wiklund, C. , & Kaitala, A. (1995). Sexual selection for large male size in a polyandrous butterfly: The effect of body size on male versus female reproductive success in *Pieris* *napi* . Behavioral Ecology, 6, 6–13.

