# Supplementary material for: Population‐specific responses of an insect herbivore to variation in host‐plant quality
Source: Ecol Evol. 2021 Nov 24;11(24):17963–72. doi: 10.1002/ece3.8392 (PMC8717263; doi:10.1002/ece3.8392)
Supplement: Supplementary file 1 — Appendix S1 [file ECE3-11-17963-s001.docx]

**Appendix S1**


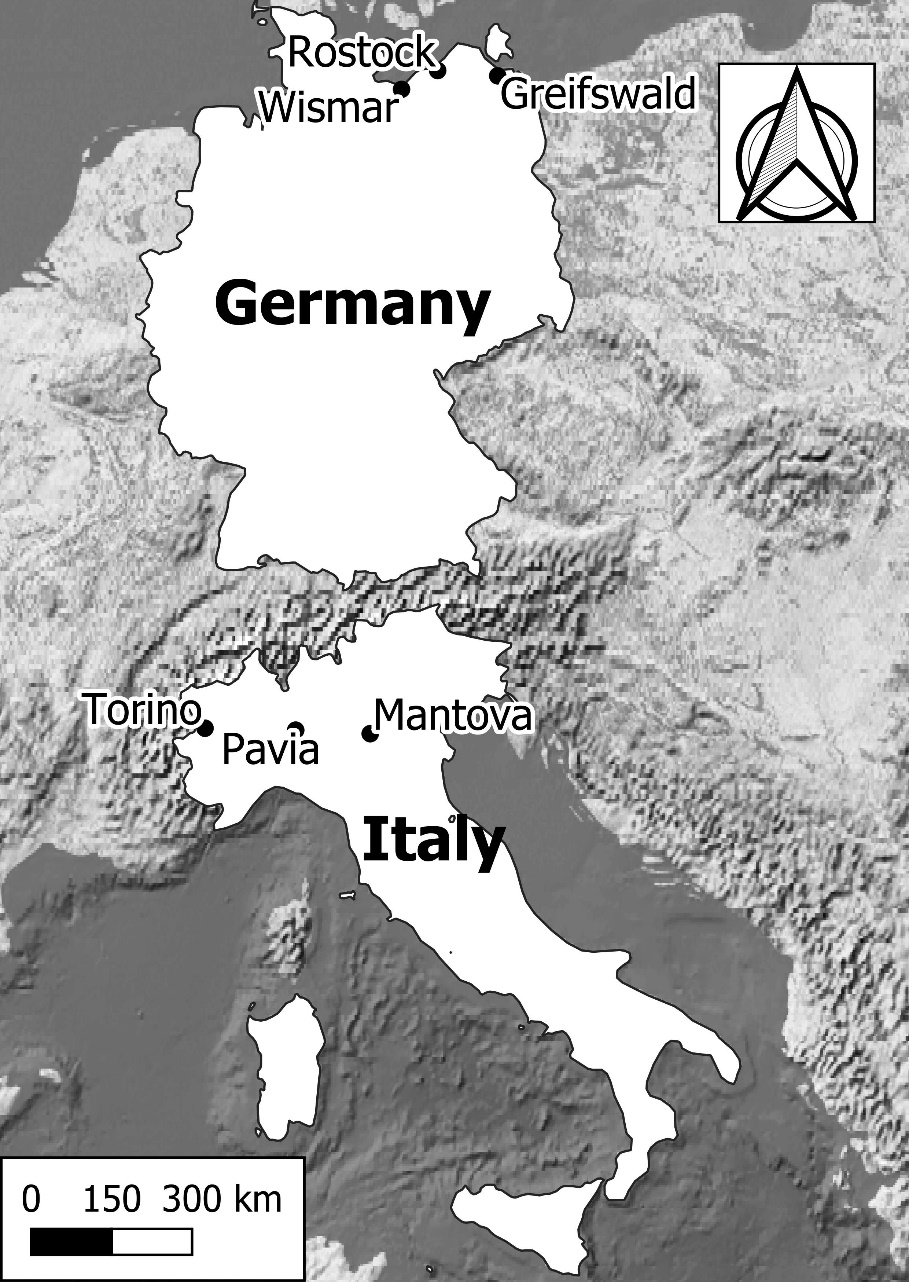


**Fig. S1** Sampling locations of *Pieris napi* populations collected in Germany (Greifswald, Rostock and Wismar) and in Italy (Mantova, Pavia and Torino). Base map from Natural Earth (<https://www.naturalearthdata.com/>, last accessed on 21.10.2021).


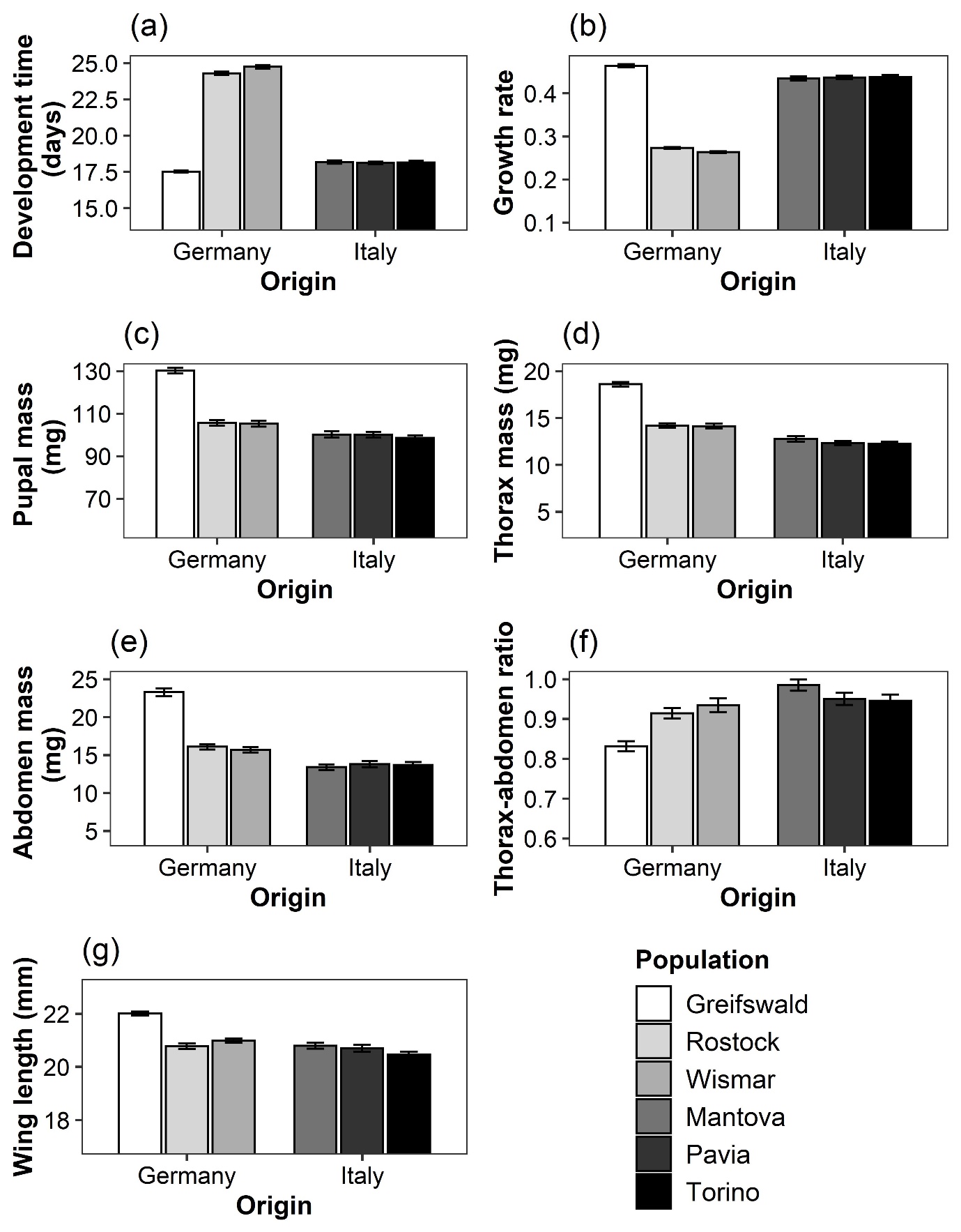


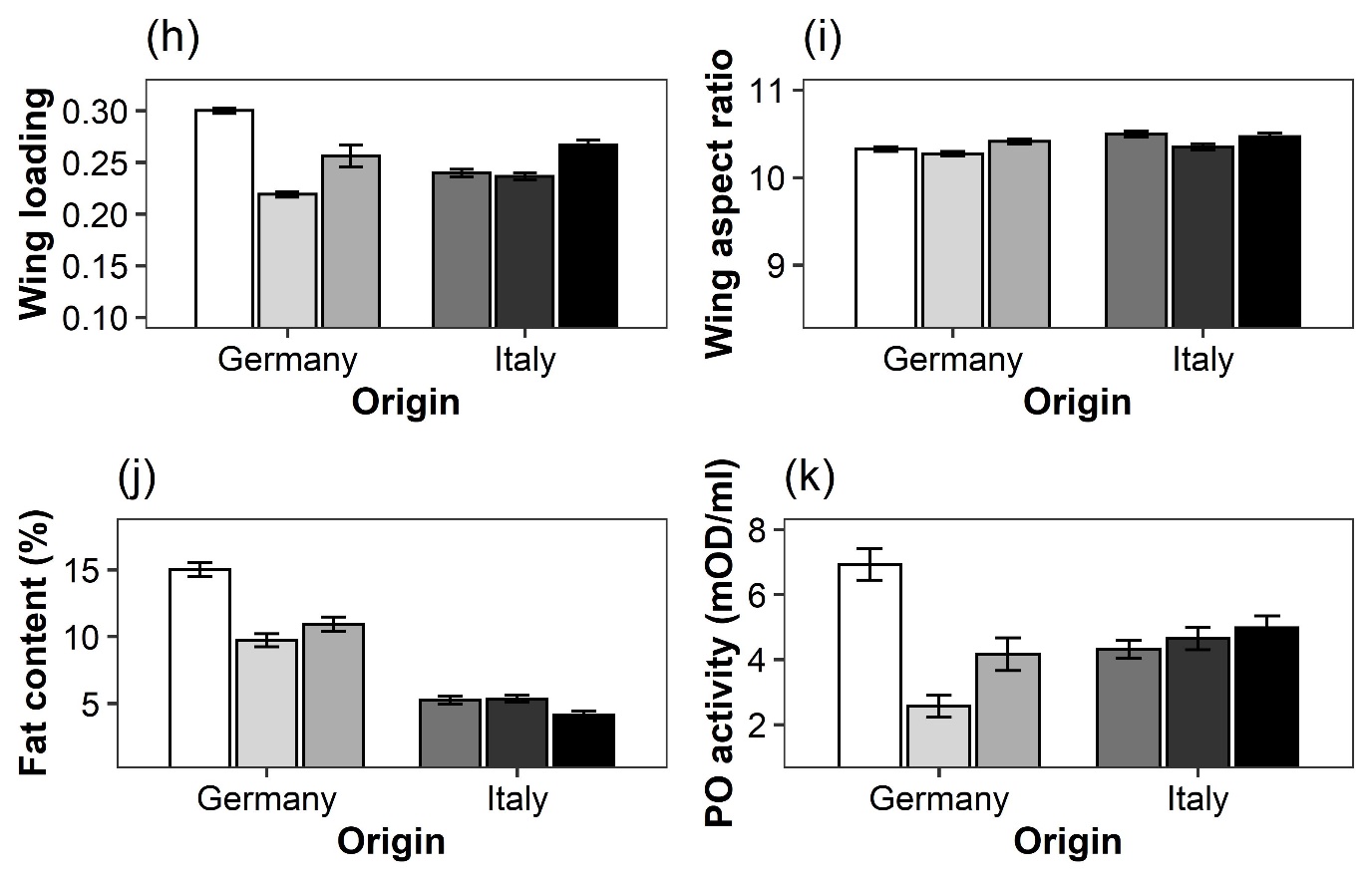


**Fig. S2** Effects of origin (Germany vs. Italy) and population (Greifswald, Rostock, Wismar, Mantova, Pavia, Torino) on (a) development time, (b) larval growth rate, (c) pupal mass, (d) thorax mass, (e) abdomen mass, (f) thorax-abdomen ratio, (g) wing length, (h) wing loading, (i) wing aspect ratio, (j) fat content and (k) phenoloxidase activity in *Pieris napi*. Given are means ± SE.
